# Neonatal anthropometry and neonatal outcome

**DOI:** 10.1590/S1516-31802003000400001

**Published:** 2003-07-01

The use of anthropometric references for the evaluation of intrauterine growth has shown that children whose growth was restricted are more predisposed to metabolic disturbances during the neonatal period, alterations in somatic and neurocognitive development during infancy,^1^ increased morbidity and mortality in the first years of life^2^ and the appearance of chronic non-transmissible diseases during adulthood.^3^

Birth weight has been the index most used for assessment of intrauterine growth. Weight is not an ideal measurement for evaluating growth: it is merely easier to measure, and the balances now available can do this to a precision of five grams. The weight gives an assessment of all the tissues together, and greater weight does not necessarily signify good growth: it may be achieved at the cost of liquid retention or fat deposition.^4^ On the other hand, the measurement of length requires a refined technique, with an appropriate instrument, and it is not always easy among newborns. Other measurements and the relationships between these have been utilized in such evaluations: cranial perimeter,^5^ skinfolds,^6^ mid-arm circumference in association with cephalic perimeter and skinfolds,^7^ and mid-arm circumference and muscle area.^8^ All these measurements have technical or interpretative limitations on their results, which makes it difficult to use them routinely in clinical practice.

The evaluation of weight distribution at birth is more adequate when it is associated with gestational age, as has been widely done since Lubchenco (1963)^9^ published his intrauterine growth curves. Methodological differences and the prevalence of factors known to be associated with reduced birth weight may explain the large differences seen between this and other curves developed subsequently.^10-12^

The observation that not all babies born with low weight are born before the full gestational term led, in the words of Wilcox (2001),^13^ to the invention of a new disease from the 1970s onwards: intrauterine growth restriction (IUGR). The usual definition of IUGR is "small for the gestational age", i.e. the smallest 10% of each stratum of gestational age.

The determination of gestational duration is also a problem in daily clinical practice. It is usually obtained via information from the mother regarding the date of the last menstruation, which brings the possibility of various orders of error, from the women who cannot remember the date to those who present menstrual irregularity or menstrual bleeding even after some time has passed since the start of the pregnancy. On the other hand, ultrasound, which is considered to be the gold standard for this evaluation when performed early in the pregnancy,^14^ is not always widely available in developing countries. In addition to this, its utilization is not totally independent of the date of the last menstruation, given that this is the basis for settling when the first ultrasound should be performed.

Thus, an index for quantifying body mass that takes into account the weight and considers the influence of the length at birth, and which is independent of gestational age, would be considered more suitable for the evaluation of the appropriateness of the child's size at birth.^15^ The ratio of weight for height can be used for measuring obesity, because if the weight is corrected by the height, high levels of this index should then be encountered among individuals that are more obese.^16^ Khosla and Lowe (1967)^17^ established that small individuals do not have a greater probability of being obese than tall people and that, for an index constructed from weight and length to translate obesity, it should satisfy the following criteria: (1) high correlation with weight; (2) distribution independent of height. Gasser et al (1994)^18^ expanded these criteria: (3) it must be correlatable with skinfolds; (4) the dependence on age and sex must not be very accentuated; (5) it must have normal distribution or accept a transformation that makes it follow a normal distribution. Several authors have used these criteria for evaluating some well-known indices, such as: *weight*/ *length*, *weight/length-squared* (Quetelet index) and *weight/ length-cubed* (ponderal index). For adults, Florey (1970)^16^ considered that the greatest correlation with height was presented by the ponderal index, and this was considered to be the least suitable as a measure of obesity. Rolland-Cachera et al. (1982)^19^ tested the validity of the three indices on healthy children aged from one month to 16 years old, and found greater correlation of *weight/length-squared* with skinfolds, and considered this to be the most valid for estimating obesity in this group. Patterson & Pouliot (1987)^20^ reported greater association of the ponderal index with perinatal morbidity (evaluated in terms of Apgar less than seven at five minutes of life, aspiration of meconium or polycythemia) than the birth weight. Cole et al. (1995)^21^ proposed the use of reference curves for the Quetelet index, together with weight and height curves, for assessing size and shape within the 0 to 23-year-old age group.

Neonatologists have preferentially used the ponderal index when there is a need to use indices that involve weight and length. The indices proposed in the study presented in this issue of the Journal^22^ have the advantage of dispensing with information on the gestational age and have been shown to be strong predictors of neonatal disease risk. Moreover, the fact that the weight/length index proposed represents factors associated not only with IUGR but also with preterm birth may represent an advantage in relation to the ponderal index, since it is not considered to be an appropriate measurement for intrauterine malnutrition, when the gestational duration is taken into account.

## References

[1] Leitner Y, Fattal-Valevski A, Geva R (2000). Six-year follow-up of children with intrauterine growth retardation: long-term, prospective study. J Child Neurol.

[2] Morris SS, Victora CG, Barros FC (1998). Length and ponderal index at birth: associations with mortality, hospitalizations, development and post-natal growth in Brazilian infants. Int J Epidemiol.

[3] Barker DJP (1998). Mothers, babies and health in later life.

[4] Moreira MEL (1997). Avaliação do crescimento e da composição corporal de recém-nascidos pré-termo [thesis].

[5] Brandt I, Falkner F, Tanner JM (1986). Growth dynamics of low birth weight infants with emphasis on the perinatal period. Human growth: a comprehensive treatise.

[6] Dauncey MJ, Gandy G, Gairdner D (1977). Assessment of total body fat in infancy from skinfold thickness measurements. Arch Dis Child.

[7] Sasanow SR, Georgieff MK, Pereira GR (1986). Mid-arm circumference and mid-arm/head circumference ratios: standard curves for anthropometric assessment of neonatal nutritional status. J Pediatr.

[8] Frisancho AR (1974). Triceps skin fold and upper arm muscle size norms for assessment of nutrition status. Am J Clin Nutr.

[9] Lubchenco LO, Hansman C, Dressler M, Boyd E (1963). Intrauterine growth as estimated from liveborn birth-weight data at 24 to 42 weeks of gestation. Pediatrics.

[10] Williams RL, Creasy RK, Cunningham GC, Hawes WE, Norris FD, Tashiro M (1982). Fetal growth and perinatal viability in California. Obstet Gynecol.

[11] Zhang J, Bowes WA (1995). Birth-weight-for-gestational-age patterns by race, sex, and parity in the United States population. Obstet Gynecol.

[12] Alexander GR, Himes JH, Kaufman RB, Mor J, Kogan M (1996). A United States national reference for fetal growth. Obstet Gynecol.

[13] Wilcox AJ (2001). On the importance – and the unimportance – of birthweight. Int J Epidemiol.

[14] Alexander GR, Allen MC (1996). Conceptualization, measurement, and use of gestational age. I. Clinical and public health practice. J Perinatol.

[15] Andréa M (1999). Regiões de tolerância para algumas medidas antropométricas do recémnascido na área urbana de Ribeirão Preto: uma aplicação [thesis].

[16] Florey Cdu V (1970). The use and interpretation of ponderal index and other weight-height ratios in epidemiological studies. J Chronic Dis.

[17] Khosla T, Lowe CR (1967). Indices of obesity derived from body weight and height. Br J Prev Soc Med.

[18] Gasser T, Ziegler B, Seifert B, Prader A, Molinari L, Largo R (1994). Measures of body mass and of obesity from infancy to adulthood and their appropriate transformation. Ann Hum Biol.

[19] Rolland-Cachera MF, Sempé M, Guilloud-Bataille M, Patois E, Péquignot-Guggenbuhl F, Fautrad V (1982). Adiposity indices in children. Am J Clin Nutr.

[20] Patterson RM, Pouliot MR (1987). Neonatal morphometrics and perinatal outcome: who is growth retarded?. Am J Obstet Gynecol.

[21] Cole TJ, Freeman JV, Preece MA (1995). Body mass index reference curves for the UK, 1990. Arch Dis Child.

[22] Bertagnon JRD, CAM Segre, Dall Colletto GM (2003). Weight-for-length relationship at birth to predict neonatal diseases. São Paulo Med J.

[23] Cole TJ, Henson GL, Tremble JM, Colley NV (1997). Birthweight for length: ponderal index, body mass or Benn index?. Ann Hum Biol.

